# Supplementary material for: Contrasting evolutionary responses in two co-distributed species of Galaxias (Pisces, Galaxiidae) in a river from the glaciated range in Southern Chile
Source: R Soc Open Sci. 2020 Jul 8;7(7):200632. doi: 10.1098/rsos.200632 (PMC7428232; doi:10.1098/rsos.200632)
Supplement: Supplementary Figures and Tables [file rsos200632supp1.docx]

Electronic Supplementary Material

Supplementary tables and figures for the article:

**Contrasting evolutionary responses in two codistributed species of Galaxias (Pisces, Galaxiidae) in a river from the glaciated range in Southern Chile**

**Submitted to the Royal Society Open Science**

Victoriano PF , Muñoz-Ramírez CP, Canales-Aguirre CB, Jara A, Iván Vera-Escalona, Burgos-Careaga T, Muñoz-Mendoza C, Habit EM

Table S1: Locality data and number of *G. maculatus* and *G. platei* individuals sampled in the River Valdivia.

|  | Zone | Site | Latitude | Longitude | ***G. maculatus*** | ***G. platei*** |
| --- | --- | --- | --- | --- | --- | --- |
| 1 | Z4 | Puente Cutipay | -39.855 | -73.333 | 9 |  |
| 2 | Z4 | Huellelhue | -39.789 | -73.125 | 6 | 4 |
| 3 | Z4 | Arique | -39.811 | -73.040 |  | 1 |
| 4 | Z4 | Calle Calle | -39.788 | -73.015 | 33 |  |
| 5 | Z4 | San Javier | -39.788 | -72.960 |  | 5 |
| 6 | Z4 | La Quinta | -39.763 | -72.884 | 2 |  |
| 7 | Z3 | Quinchilca | -39.854 | -72.755 | 8 | 1 |
| 8 | Z3 | Camping | -39.853 | -72.752 | 1 | 5 |
| 9 | Z3 | El Mosqueto | -39.813 | -72.726 | 15 | 15 |
| 10 | Z3 | Balsa Sur | -39.804 | -72.724 | 7 | 2 |
| 11 | Z3 | Balsa Norte | -39.801 | -72.701 | 10 | 3 |
| 12 | Z3 | Anita Maria | -39.772 | -72.697 | 1 | 4 |
| 13 | Z3 | Cun Cun Oeste | -39.749 | -72.630 | 1 | 5 |
| 14 | Z3 | Nuevo Maiten | -39.760 | -72.589 | 2 |  |
| 15 | Z3 | Represa | -39.760 | -72.582 | 2 | 1 |
| 16 | Z3 | Forestal Maiten | -39.759 | -72.573 | 3 |  |
| 17 | Z3 | Forestal Tornagalones | -39.749 | -72.493 |  | 1 |
| 18 | Z2 | Los Bajos | -39.759 | -72.468 | 9 | 4 |
| 19 | Z2 | Piedra Blanca | -39.772 | -72.460 | 23 | 18 |
| 20 | Z2 | Desembocadura Catalina | -39.776 | -72.459 | 1 |  |
| 21 | Z2 | Hotel (Lago Riñihue) | -39.775 | -72.456 | 15 | 18 |
| 22 | Z2 | Playa Choshuenco | -39.783 | -72.404 | 12 | 1 |
| 23 | Z2 | Desembocadura Enco | -39.902 | -72.168 | 1 | 3 |
| 24 | Z2 | Rio Blanco | -39.912 | -72.159 | 2 |  |
| 25 | Z1 | Puente Rucatrehue | -39.751 | -72.151 |  | 12 |
| 26 | Z1 | Lago Panguipulli | -39.783 | -72.094 | 1 | 2 |
| 27 | Z1 | Puente Paullahuente | -39.820 | -72.082 | 1 |  |
| 28 | Z1 | Fuy - Punahue | -39.827 | -72.042 | 3 | 1 |
| 29 | Z1 | Rio Llanquihue | -39.839 | -72.031 | 8 |  |
| 30 | Z1 | Lago Neltume | -39.768 | -71.967 | 15 | 3 |
| 31 | Z1 | Puente Quilmio | -39.814 | -71.885 | 9 |  |
| 32 | Z1 | Puente Pullinque | -39.597 | -72.218 | 13 | 5 |
| 33 | Z1 | Lago Calafquen | -39.580 | -72.021 | 7 |  |
| 34 | Z1 | Puente Pellaifa | -39.588 | -72.016 | 5 | 22 |
| Total |  |  |  |  | 225 | 136 |


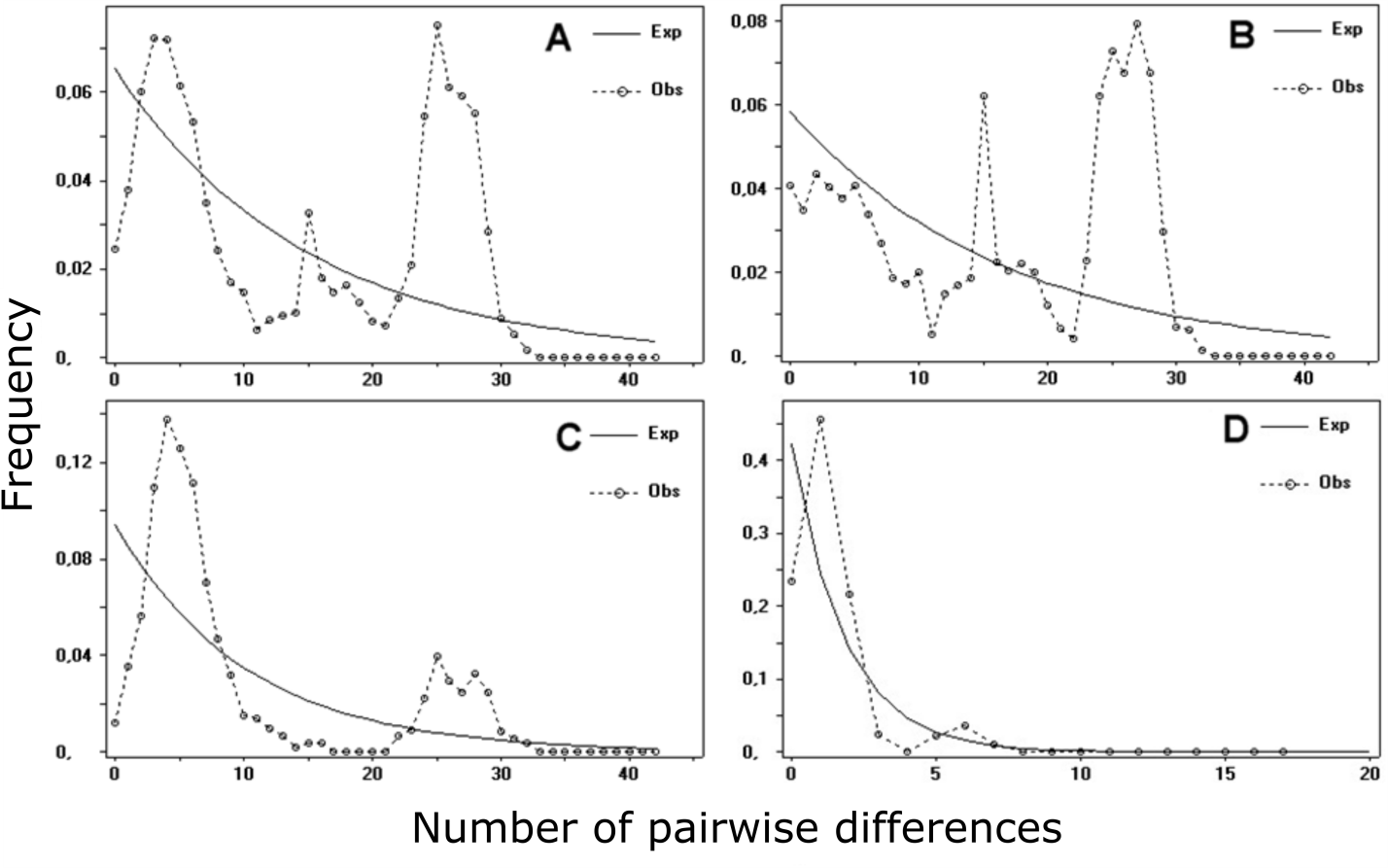


Figure S1: Mismatch distribution for A: *G. maculatus* for the total study area, B: *G. maculatus* from non-glaciated areas, C: *G. maculatus* from glaciated areas, D: *G. platei* from the total study area.


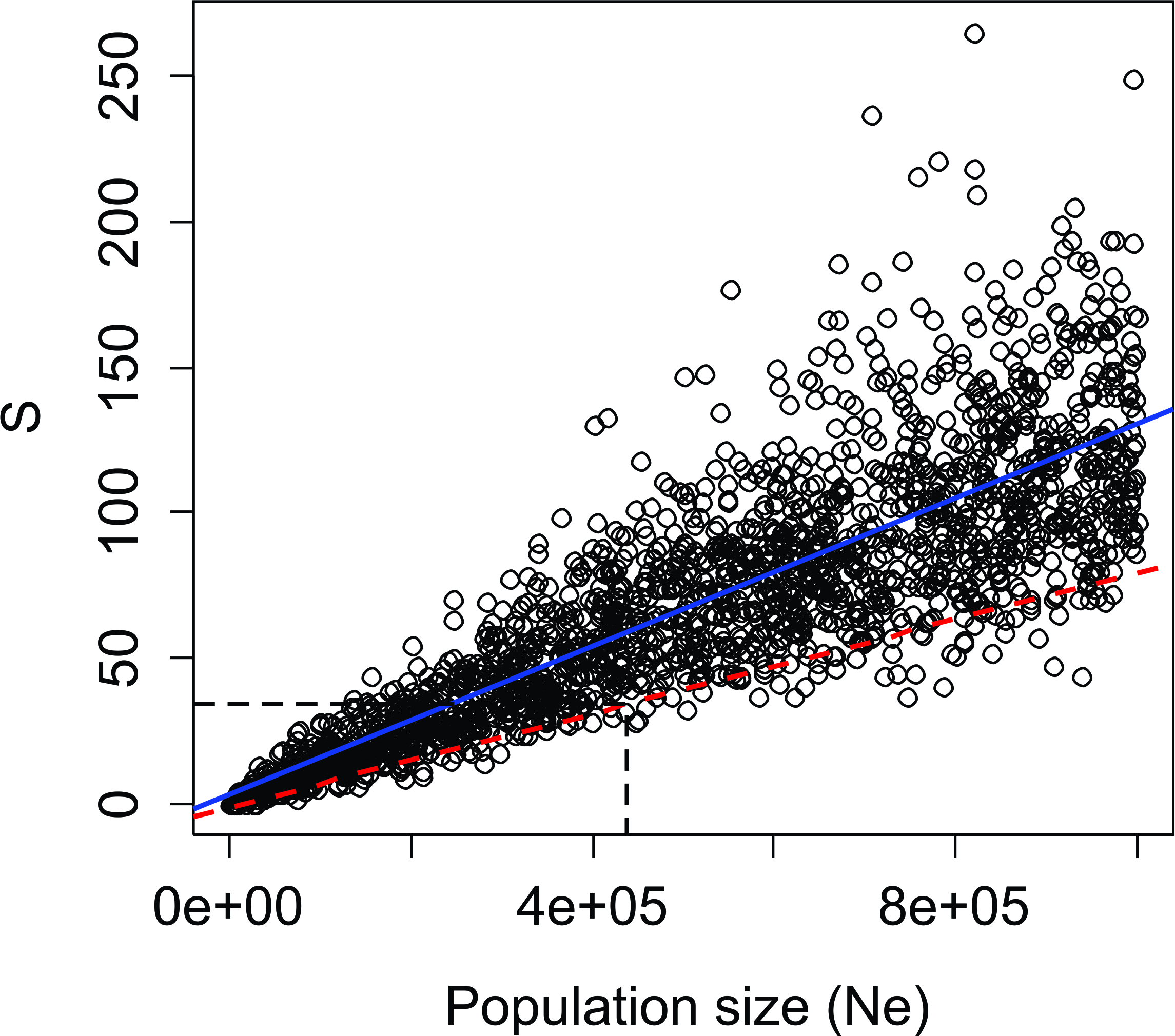

Figure S2: Results from a first round of 1000 simulations used to select an appropriate Ne-value for subsequent genetic simulations. The y-axis represents the variation of the number of segregating sites (S) calculated from each simulation as a function of population size (Ne). The blue line represents the best-fit regression line, whereas the red dashed line represents the 5% lower quantile regression line.


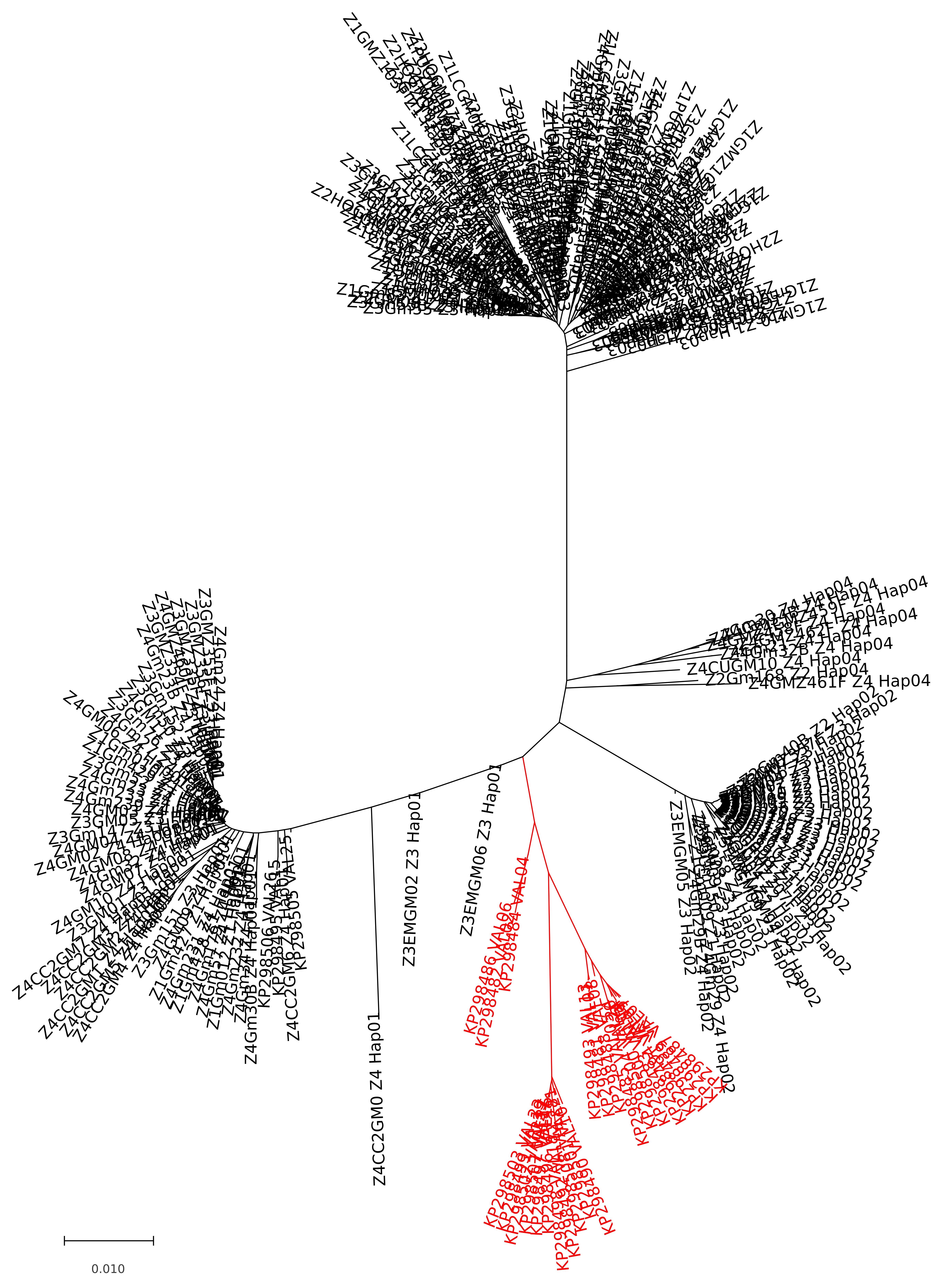


Figure S3: Neighbor joining tree for *Galaxias maculatus* based on our 225 D-Loop sequences plus 28 D-Loop sequences from González-Wevar et al. (2015) (GenBank acc. numbers KP298481-KP298508), shown in red. The tree was built in Mega X (Kumar et al. 2018). Samples from Gonzalez-Wevar et al. (2015) are from offshore the Valdivia River mouth and form a monophyletic clade distinct from all other samples upstream the Valdivia Basin.

References

González-Wevar CA, Salinas P, Hüne M, Segovia NI, Vargas-Chacoff L, Astorga M, Cañete JI, Poulin E. 2015 Phylogeography in Galaxias maculatus (Jenyns, 1848) along Two Biogeographical Provinces in the Chilean Coast. PLoS One 10, e0131289.

Kumar S., Stecher G., Li M., Knyaz C., and Tamura K. (2018). MEGA X: Molecular Evolutionary Genetics Analysis across computing platforms. Molecular Biology and Evolution 35:1547-1549.
